# Antioxidant and Photoprotective Activity of Apigenin and Its Potassium Salt Derivative in Human Keratinocytes and Absorption in Caco-2 Cell Monolayers

**DOI:** 10.3390/ijms20092148

**Published:** 2019-04-30

**Authors:** Noelia Sánchez-Marzo, Almudena Pérez-Sánchez, Verónica Ruiz-Torres, Adrián Martínez-Tébar, Julián Castillo, María Herranz-López, Enrique Barrajón-Catalán

**Affiliations:** 1 Instituto de Biología Molecular y Celular (IBMC) and Instituto de Investigación, Desarrollo e Innovación en Biotecnología Sanitaria de Elche (IDiBE), Universitas Miguel Hernández, 03202 Elche, Spain; n.sanchez@umh.es (N.S.-M.); almudena.perez@umh.es (A.P.-S.); vruiz@umh.es (V.R.-T.); mherranz@umh.es (M.H.-L); 2 Programs of Molecular Mechanisms and Experimental Therapeutics in Oncology (ONCOBell), Catalan Institute of Oncology, Bellvitge Institute for Biomedical Research, Granvia de l’Hopitalet 199, 08908, L’Hospitalet de Llobregat, 08907 Barcelona, Spain; amartinezt@idibell.cat; 3 Nutrafur S.A., Camino Viejo de Pliego, km.2, 30820 Alcantarilla, Murcia, Spain; j.castillo@nutrafur.com

**Keywords:** flavonoid, apigenin, photoprotection, UV radiation, antioxidant, keratinocytes, absorption

## Abstract

Ultraviolet (UV) radiation, especially types A (UVA) and B (UVB), is one of the main causes of skin disorders, including photoaging and skin cancer. Ultraviolent radiation causes oxidative stress, inflammation, p53 induction, DNA damage, mutagenesis, and oxidation of various molecules such as lipids and proteins. In recent decades, the use of polyphenols as molecules with an antioxidant and anti-inflammatory capacity has increased. However, some of these compounds are poorly soluble, and information regarding their absorption and bioavailability is scarce. The main objective of this study was to compare the intestinal absorption and biological activity of apigenin and its more soluble potassium salt (apigenin-K) in terms of antioxidant and photoprotective capacity. Photoprotective effects against UVA and UVB radiation were studied in human keratinocytes, and antioxidant capacity was determined by different methods, including trolox equivalent antioxidant capacity (TEAC), ferric reducing antioxidant power (FRAP) and oxygen radical absorbance capacity (ORAC) assays. Finally, the intestinal absorption of both apigenins was determined using an in vitro Caco-2 cell model. Apigenin showed a slightly higher antioxidant capacity in antioxidant activity assays when compared with apigenin-K. However, no significant differences were obtained for their photoprotective capacities against UVA or UVB. Results indicated that both apigenins protected cell viability in approximately 50% at 5 J/m^2^ of UVA and 90% at 500 J/m^2^ of UVB radiation. Regarding intestinal absorption, both apigenins showed similar apparent permeabilities (*P_app_*), 1.81 × 10^−5^ cm/s and 1.78 × 10^−5^ cm/s, respectively. Taken together, these results suggest that both apigenins may be interesting candidates for the development of oral (nutraceutical) and topical photoprotective ingredients against UVA and UVB-induced skin damage, but the increased water solubility of apigenin-K makes it the best candidate for further development.

## 1. Introduction

The skin is the largest organ of the body acting as a physical and chemical barrier to protect the body against harmful external agents such as ultraviolet (UV) radiation, dehydration, temperature changes, and pathogens [1,2,3]. Furthermore, the skin is a sensitive organ because the nerve endings and receptors related to the sense of touch and temperature are located within the skin. UV radiation exposure is a main factor for age-related changes, including immunosuppression and allergy disorders, degenerative aging, inflammation, extracellular matrix (ECM) degeneration, DNA damage, oxidative stress, and carcinogenesis [4,5]. These effects are included in the term photoaging, which resumes the biological consequences of UV exposure, not only on skin, but also in the whole organism.

UV radiation is divided into (a) long-wave UVA (320–400 nm), (b) medium-wave UVB (280–320 nm), and (c) short-wave UVC (100–280 nm) radiation, of which UVC is absorbed by the ozone layer [4]. UVA comprises more than 90% of all solar UV radiation that reaches the Earth’s surface and is considered the “aging ray” that penetrates the epidermis and dermis [6]. UVA enhances the expression of metalloproteinases (MMPs) that lead to collagen decrease and abnormal elastic fiber overgrowth, resulting in stratum corneum thickening and epidermal hyperplasia [7,8]. Moreover, UVB is a minor component of sunlight that is capable of producing direct changes on biomolecules, causing the acute effects of sunlight exposure, such as erythema (sunburn) or pigmentation (tanning). It is well established that UVB photons target DNA causing dimeric photoproducts between adjacent pyrimidine bases to form [9]. The incorrect repair of these photolesions can affect tumor suppressor genes and oncogenes, and therefore, UVB is the main radiation associated with melanoma and nonmelanoma skin cancer risk. Nonetheless, it has been suggested that UVA has mutagenic and carcinogenic actions through the generation of reactive oxygen species (ROS), which also damage DNA [10]. In addition, excessive ROS formation, not only after UVA but also following UVB overexposure, can oxidize several DNA repair proteins, compromising their efficiency [11].

In recent years, there have been a large number of studies and publications related to natural extracts and compounds that exert beneficial effects for human health [4], especially polyphenols [12]. These compounds are widely distributed in fruits, vegetables, and legumes, as well as in red wine and tea [13,14]. Polyphenols are divided into the phenolic acids, stilbenes, flavonoids, and lignans [15] (Supplementary Figure S1A). Their structural diversity is the reason why polyphenols present different biological functions, such as antitumor [15,16], anti-inflammatory [17], antimicrobial [18], antioxidant [19], and photoprotective activities [20,21,22]. Furthermore, prospective studies have reported that polyphenols have the ability to prevent cardiovascular diseases [23,24].

The flavonoid group represents 60% of the total natural polyphenols and can be classified into different subgroups (Supplementary Figure S1A). In recent years, flavonoids have generated great interest because they are the major group of polyphenols present in the human diet [25,26]. Pure flavonoids are quite insoluble in water, making this characteristic their main drawback when both studying their biological activity and using them on a new nutraceutical, cosmeceutical and/or pharmaceutical formulation. There are different strategies to solve this water insolubility problem. Plants usually glycosylate flavonoids to enhance their solubility or to store them in vacuoles [27,28]. Another strategy, which is especially relevant for industrial purposes, is to derivate these compounds by obtaining salt species with an improved water solubility profile [29]. Solubility is a relevant property in drug development, but drug absorption must not be forgotten. In fact, the actual Biopharmaceutics Classification System (BCS) is based on both solubility and absorption data, allowing researchers and pharmaceutical companies to predict in vivo bioavailability based on in vitro data [30].

One of the most known and studied flavonoids is 4′,5,7-trihydroxyflavone, commonly known as apigenin (Supplementary Figure S1B). This bioactive compound is found in many fruits and vegetables, such as chamomile flowers, thyme, onions, and spices [13]. Several studies have shown that this natural compound has potential anti-cancer activity [31], antioxidant and anti-inflammatory effects [32], and antimicrobial activity [33] (Supplementary Figure S1B). However, it exhibits poor solubility in water, hampering the in vitro dissolution rate, efficacy and oral bioavailability [34]. This situation also hinders in vitro studies, as most assays are developed in an aqueous environment using cell culture assays that require the compounds to be soluble enough to be used. To avoid this situation, different apigenin salt derivates were obtained, leading to increased solubility [30,34]. However, there have been no comparative works studying whether this salt derivatization influences biological activity except for one covering angiotensin-converting enzyme inhibitory activity from a structural point of view and comparing this activity with that of a collection of other non-salt-derived flavonoids [35].

In the present study, apigenin flavone and its more water-soluble potassium salt derivative (apigenin-K) were compared to determine their antioxidant capacity, their inhibition of UVA and UVB harmful effects on human keratinocytes, and their protection against DNA damage. In addition, the apparent permeability of both apigenins was studied in an in vitro model of intestinal absorption using the Caco-2 cell line.

## 2. Results

### 2.1. Antioxidant Activity

A set of antioxidant assays was carried out to determine the antioxidant potential of apigenin and apigenin-K (Table 1). The single-electron transfer-based methods Trolox equivalent antioxidant capacity (TEAC) and ferric reducing antioxidant power (FRAP) were performed. These methods are widely used in a large variety of food and biological samples [36,37]. For the TEAC assay, no significant differences were obtained. For the FRAP assay, apigenin and apigenin-K exhibited slight but significant differences (Table 1) between them. In addition, the oxygen radical absorbance capacity (ORAC) assay, based on a hydrogen atom transfer mechanism, was used to measure the capacity to eliminate peroxyl radicals. Values for apigenin and apigenin-K revealed a high oxygen radical absorbance capacity for both compounds with small, but significant differences (Table 1).

The absorption spectra were compared for both apigenins, showing no differences between them and both presenting the typical flavone absorption maxima at 270 and 340 nm [38]. These absorption maxima partially match the UVA and UVB ranges.

### 2.2. Apigenin and Apigenin-K Protect Human Keratinocytes against UVA and UVB Radiation

**Figure 1:**
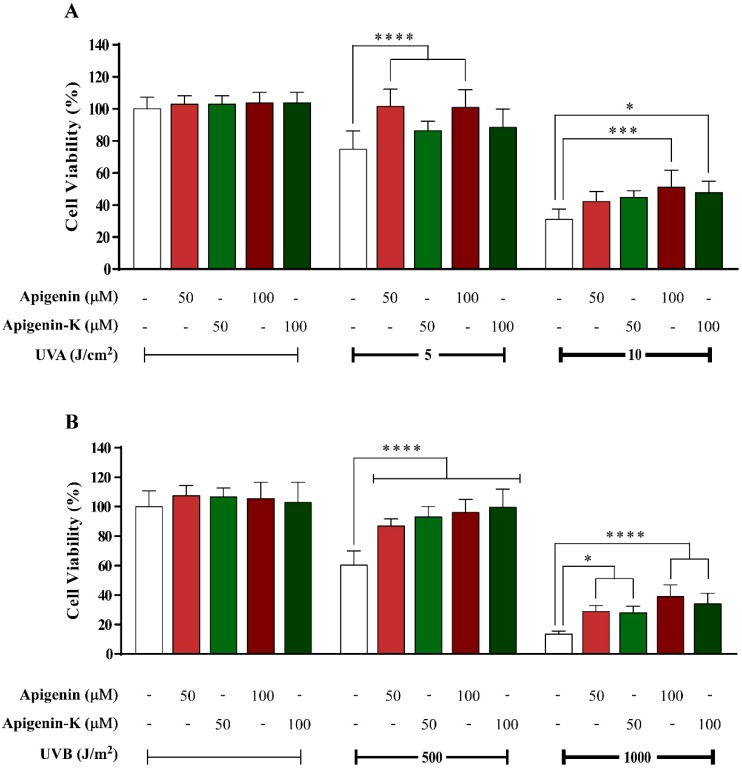
Human keratinocyte survival after irradiation with a 5 J/cm^2^ or 10 J/cm^2^ dose of UVA (**A**) or 500 J/m^2^ or 1000 J/m^2^ dose of UVB (**B**), in the presence of apigenin or apigenin-K (50 or 100 µM), was determined using the MTT assay after the incubation of cells for 72 h post-irradiation. The data are expressed as the means of 6 replicates ± SDs. * *p* < 0.05, *** *p* < 0.001 and **** *p* < 0.0001 indicate statistically significant differences compared with the irradiated sample in the absence of compound.

Apigenin and apigenin-K were tested to determine their capacity to protect human keratinocytes from UVA- and UVB-induced damage at different radiation doses (5 or 10 J/cm^2^ and 500 or 1000 J/m^2^, respectively). Photoprotection percentage was calculated according to Equation (1), showed in Materials and Methods section. After cell irradiation with 5 J/cm^2^ UVA, both apigenins showed over 50% protection when compared with control cells irradiated with the same UVA dose in the absence of compound (Figure 1A). While there were no differences between equimolar concentrations of each apigenin or between different concentrations of the same apigenin, apigenin (maroon bars) showed statistically significant differences when compared with the control (white bar). When the UVA dose was increased up to 10 J/cm^2^, cell viability was reduced accordingly. Once again, no differences were observed between equimolar concentrations of each apigenin or between different concentrations of the same apigenin; however, significant differences were obtained for the highest concentration of each compound when compared with control cells (Figure 1A). Similar results were obtained after UVB irradiation with 500 and 1000 J/m^2^, as both compounds exerted statistically significant photoprotection versus control cells, also with a dose-response behavior (Figure 1B).

### 2.3. Determination of Apparent Permeability (Papp) Values

**Figure 2:**
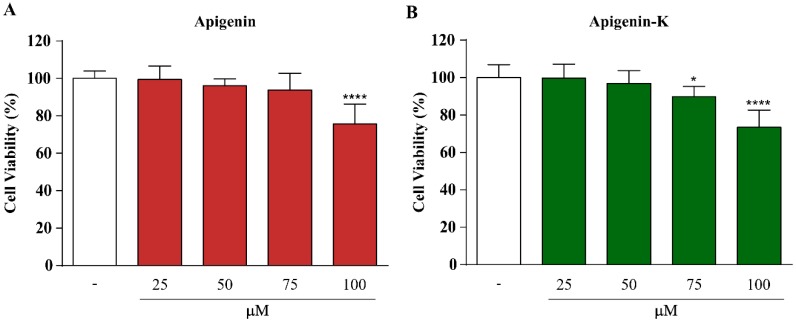
Cytotoxic effects of apigenin (**A**) and apigenin-K (**B**) treatment (25, 50, 75 or 100 µM) for 2 h in Caco-2 cells determined using the MTT assay. The data are expressed as the means of 6 replicates ± SDs. * *p* < 0.05 and **** *p* < 0.0001 indicate statistically significant differences compared with nontreated cells.

Caco-2 cells are a well-established in vitro model for the investigation of intestinal permeability of different compounds or drugs [34,35,36]. The optimum concentration of apigenin and apigenin-K was determined using the MTT assay before permeability studies to prevent monolayer damage. Figure 2 shows statistically significant cytotoxicity only at a concentration of 100 µM for both apigenins. However, a viability decrease was observed when Caco-2 cells were treated with 75 µM of each apigenin. Therefore, a final concentration of 50 µM was used in the transport experiments.

**Figure 3:**
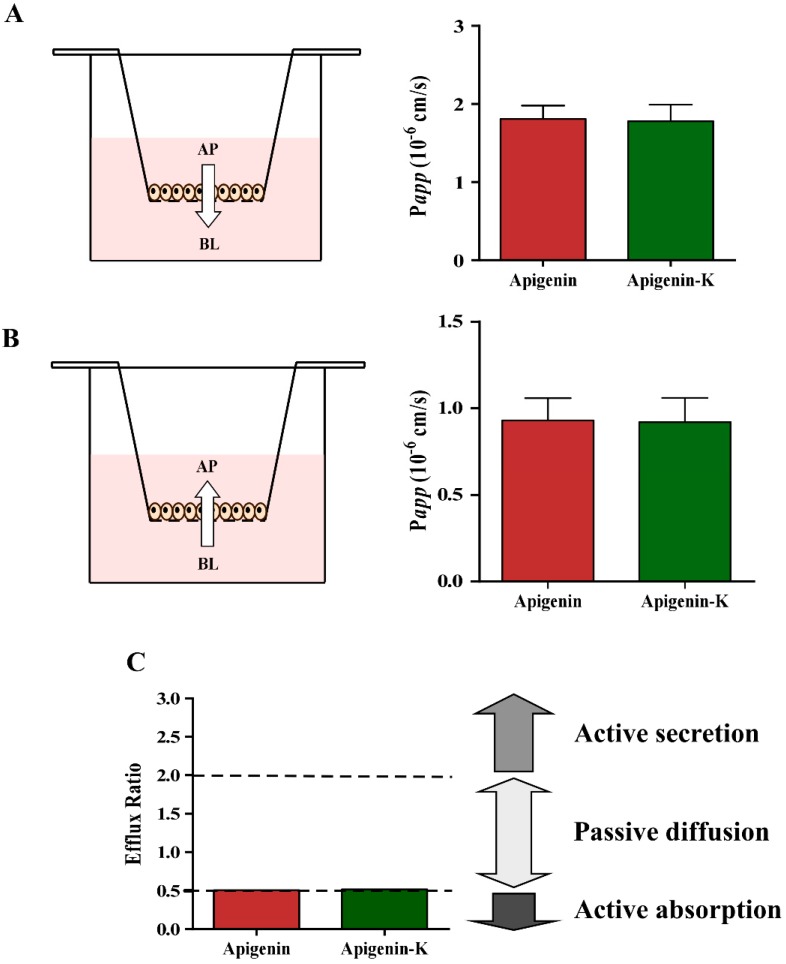
*P_app_* values and diagram of permeation flow for both apigenins in AP-BL (**A**) and BL-AP (**B**) directions respectively. (**C**) Efflux ratio plot for both apigenins, showing the different transport mechanism categories.

The transport across the Caco-2 cell monolayer model was monitored for a period of 120 min, as described in the Materials section, and the TEER values did not drastically change during the assay, maintaining above the level of 300–400 Ω/cm^2^ for all monolayers. Concentration values from each timepoint were determined by HPLC and were used to obtain the bidirectional *P_app_* values according to Equation (2), shown in the Materials and Methods section. Results are shown in Table 2 and Figure 3A,B. The estimated apparent permeability of both apigenins was similar in the apical (AP) to basolateral (BL) direction. Lower, but also similar, values were obtained when BL-AP transport was assayed. Moreover, the ratios of *P_app_* (BL-AP) to *P_app_* (AP-BL) were calculated to estimate the absorption mechanism using Equation (3) as described in Materials and Methods section (Table 2 and Figure 3C). This value tentatively indicates an active transport when is lower than 0.5, a passive diffusion transport when it is between 0.5 and 2 and an active secretion mechanism when it is higher than 2.

## 3. Discussion

UV radiation has an array of harmful effects, and chronic exposure to sunlight is the main cause of photoaging and skin carcinogenesis. Novel approaches for skin protection have gained scientific interest in diminishing UV exposure consequences, cancer morbidity, and the costs associated with treatment. Several phytochemicals have shown substantial photoprotective and anticarcinogenic effects and have attracted considerable attention due to their low toxicity [39]. One of these promising phytochemicals is apigenin, of which high amounts are present in common fruits and plant species such as chamomile (*Chamaemelum nobile*), celery (*Apium graveolens*), onion (*Allium cepa*), thyme (*Thymus vulgaris*), and oregano (*Origanum vulgare*) [13,40].

In this work, the antioxidant and photoprotective abilities of apigenin flavone and its more soluble potassium salt, apigenin-K, were evaluated in a skin cell model. The role of oxidative stress in the consequences of UV radiation [10] has been demonstrated. For that reason, the antioxidant capacity of both apigenins was examined first. Apigenin showed a slightly stronger capacity to scavenge free radicals than apigenin-K in the TEAC, FRAP, and ORAC assays, but those results were significant only for the FRAP and ORAC assays. As apigenin-K has one less hydroxyl (–OH) group, these results are consistent with the antioxidant mechanism of polyphenols, which is based on the number of hydroxyl (–OH) groups [41]. Furthermore, the antioxidant activity of flavonoids depends upon the arrangement of the functional groups about the nuclear structure. Flavonoids vary in their chemical substitution around the heterocyclic oxygen ring, but a C_6_–C_3_–C_6_ carbon skeleton is characteristic of all of them, comprising three rings (A, B and C), as Supplementary Figure S1B illustrates. It is generally accepted that the presence of catechol hydroxyl groups in the B ring provides the strongest antioxidant capacity exhibited by these compounds. In addition, greater antioxidant activity for flavonoids with meta-5,7-dihydroxy arrangements in the A ring has been described, as is present in apigenin [41]. Salt formation of apigenin-K may entail the loss of a hydrogen at the 7- or 4′-hydroxyl position, so the apigenin-K reduction potential should be lower [34]. Despite this, apigenin-K antioxidant activity is higher than that described for other well-known flavonoids, such as kaempferol, hesperidin, and naringenin [41,42].

The small differences between the antioxidant capacity of both apigenins exerted in the aforementioned assays were reflected in the survival increase achieved by keratinocytes exposed to UVA and UVB radiation, where the higher photoprotection of apigenin was exhibited. However, no statistically significant differences were obtained when the same concentrations of apigenin and apigenin-K were compared, suggesting that both compounds share a similar photoprotective capacity. Regarding the putative protective mechanism, some hypotheses can be deduced from the obtained data and the current state of the art. Some polyphenols have shown the ability to rapidly reach intracellular targets, so it can be postulated that apigenin and apigenin-K may be able to act as antioxidants, scavenging the ROS that originate upon UVB and UVA radiation, such as hydroxyl radicals (^·^OH), superoxide anion radicals (O_2_^·−^) and lipoperoxyl radicals (ROO^·^) [10]. In addition, both apigenins present an absorption spectrum that matches the UVA and UVB ranges, so both effects, antioxidant capacity and UV-absorption, contribute to reducing the harmful effects of radiation on cell viability.

Intestinal absorption of apigenin and its potassium salt derivative was estimated using the human Caco-2 cell culture model for nutraceutical purposes, and no statistically significant differences were found between the apparent permeability estimated for each compound regardless of the tested direction (AP-BL or BL-AP). Apigenin is usually found in its glycosidic form in fruits and vegetables, but it has been dilucidated that glycosides are efficiently hydrolyzed to the free flavonoids in the human intestinal tract by bacterial enzymes [43]. These enzymatic changes justify the use of aglicone forms of apigenin and apigenin-K, with improved water solubility, in an attempt to enhance absorption, and can be industrially obtained without significant costs and drawbacks.

The Caco-2 cell model is widely used due to its differentiation into polarized enterocyte-like cells with an apical and basolateral surface and the presence of active transport systems. Generally, high-absorbed drugs are found to have *P_app_* values higher than 1 × 10^−5^ cm/s, whereas moderately absorbed drugs had *P_app_* values above (1–10) × 10^−6^_,_ and a *P_app_* value lower than 1 × 10^−6^ is related to poorly-absorbed drugs [44]. According to these data, both apigenins present a high absorption profile, with *P_app_* values above 1 × 10^−5^ cm/s. The values estimated in the present manuscript were similar to those reported in other studies [45,46,47] and higher than the values calculated for other flavonoids such as quercetin and its metabolites [48]. Fang et al. recently studied the relationship between the structure of thirty flavonoids and their apparent permeabilities in the Caco-2 cell model [47]. They discovered that substitution on the 3′ carbon decreases flavonoid absorption, while substitution on the adjacent 2′ or 4′ carbon (as occurs in apigenin) increases absorption. Furthermore, flavones show greater permeability than their respective flavonols; for example, the apparent permeability of apigenin is 2.5-fold higher than the estimated permeability for its respective flavonol kaempferol. The calculated efflux ratios are close to 0.5, suggesting the putative participation of active transporters. However, additional assays such as utilization of the P-glycoprotein (MDR1) inhibitor verapamil in permeability studies are required [44,49] to confirm this hypothesis, as both ratios are just in the frontier between active transport and passive diffusion mechanisms. A recent work has demonstrated that apigenin and quercetin downregulate the gene expression levels of BRCP, MRP2 and MDR1 [50], the most pharmacologically relevant ABC transporters to flavonoids, which are localized on the apical side of the intestinal epithelium [51]. The function of these transporters is to efflux compounds out of the cells and into the lumen, causing the reduction of basolateral-to-apical absorption with their downregulation, which is consistent with our results. Nevertheless, the concentration-dependent behavior of apigenin in rat duodenum and jejunum, which indicated active carrier-mediated saturable mechanism in those intestinal segments [52].

Finally, according the initial objective of the study, which was the comparison between the biological activity and absorption of both compounds, it can be concluded that although some small differences in terms of antioxidant capacity have been obtained between apigenin and apigenin-K, both the photoprotective and absorption results presented no significant differences. Moreover, according to Fick’s Law of diffusion (see Equation (4) in the Material and Methods section), the increased solubility of apigenin-K makes it more interesting for future studies, as higher concentrations can be obtained in the absorption phase, increasing the absorption flow and subsequent plasma concentration and biological effects. This last aspect allows the selection of apigenin-K as the best candidate for further pharmaceutical, nutraceutical, or cosmeceutical developments.

## 4. Materials and Methods

### 4.1. Chemicals and Reagents

Human keratinocyte cells (HaCaT, a spontaneously immortalized cell line) were obtained from Cell Lines Service GmbH (Eppelheim, Germany). Dulbecco’s Modified Eagle’s Medium (DMEM), fetal bovine serum (FBS), penicillin–streptomycin, Hank’s Balanced Salt Solution (HBSS), MEM Non-Essential Amino Acids (NEAA) Solution (100×) and 1 M HEPES were obtained from Gibco (Thermo Fisher Scientific, Waltham, MA, USA). For high-performance liquid chromatography (HPLC), all chemicals were of analytical reagent grade and were used as received. For mobile phase preparation, trifluoroacetic acid (TFA) and acetonitrile were purchased from Merck (Millipore, Darmstadt, Germany) and VWR (Barcelona, Spain), respectively. Dimethyl sulfoxide (DMSO) and the remaining reagents were purchased from Sigma-Aldrich (Steinheim, Germany). Apigenin and its monopotassium salt derivative (apigenin-K), which were 96.80% and 90.53% pure, respectively, were kindly provided by NUTRAFUR, SA—Frutarom Group (Alcantarilla, Murcia, Spain).

### 4.2. Cell Culture

HaCaT cells were cultured in high-glucose DMEM containing 10% heat-inactivated FBS, 0.1 mg/mL penicillin and 100 U/mL streptomycin. Caco-2 cells were grown in high-glucose DMEM and supplemented with 10% heat-inactivated FBS, 1% NEAA, 1% HEPES, 0.1 mg/mL penicillin, and 100 U/mL streptomycin. Both cell lines were trypsinized on the third day following purchaser instructions and were maintained in a humidified 5% CO_2_ atmosphere at 37 °C.

### 4.3. Determination of Antioxidant Capacity

Three different methods were performed to determine the antioxidant capacity of both apigenins. The Trolox equivalent antioxidant capacity (TEAC) was performed, as previously reported to establish the ABTS^·+^ scavenging ability of both compounds [21]. The TEAC of the samples was calculated from the standard curve of Trolox, and the results are expressed in micromoles of Trolox equivalents (TE) per millimole of compound. The ferric reducing antioxidant power (FRAP) assay was performed, as described elsewhere [37]. The reduction of a ferric-tripyridyltriazine complex was estimated, and the results are expressed in micromoles of Fe^2+^ per millimole of compound. To assay the capacity of the compounds to scavenge peroxyl radicals, a validated assay of the oxygen radical absorbance capacity (ORAC) method was used [53]. The ORAC assay was carried out on a microplate reader (POLAstar Omega, BMG LabTech GmbH, Offenburg, Germany) with 495 nm excitation and 520 nm emission filters to monitor fluorescein oxidation. The results are expressed in micromoles of TE per millimole of compound.

### 4.4. Apigenin and Its Potassium Salt Derivative Absorption Spectra

Absorption spectra collection was performed using a microplate reader (SPECTROstar Omega, BMG LabTech GmbH, Germany). Apigenin solutions were prepared by dissolving the compounds in DMSO at a concentration of 1 µM. The results are represented by GraphPad Prism version 6.00 software using data in the range from 260 to 400 nm at 2 nm intervals [21].

### 4.5. Photoprotection Assay

Cells were cultured in 96-well plates and maintained in complete DMEM for 24 h. At 80–90% confluence, cells were treated with phosphate-buffered saline (PBS) containing apigenin or apigenin-K at a concentration of 50 or 100 µM, followed by treatment with UVA or UVB light emitted from a Bio-Link Crosslinker BLX-E312 (Vilber Lourmat, France) at 5 or 10 J/cm^2^_,_ or 500 or 1000 J/m^2^, respectively [20]. Afterwards, the PBS was replaced with fresh medium, and the cells were incubated for 72 h. Cell viability was measured using the MTT assay. The medium was removed, and the cells were incubated with MTT for 3–5 h at 37 °C with 5% CO_2_. Then, the medium was discarded, and 100 µL of DMSO per well was added to dissolve the formazan crystals. After 15 min of shaking, the absorbance was measured using a microplate reader (SPECTROstar Omega, BGM LabTech GmbH, Offenburg, Germany) at 570 nm. The photoprotection percentage was obtained using the following formula:

(1) $$\mathrm{Photoprotection}\;\left(\%\right)=100\times 100-\frac{\left(PC-sample\right)}{\left(PC-NC\right)}$$

where *PC* is the % survival in the nonirradiated control and the nontreated and nonirradiated sample and *NC* is the % survival in the irradiated control and the nontreated but irradiated sample.

### 4.6. Permeability Studies

The cytotoxic effects of apigenin and apigenin-K were tested using the MTT assay. Caco-2 cells were seeded in 96-well plates until cell monolayers were obtained. Then, the cells were treated with different concentrations of apigenin and apigenin-K (25, 50, 75, or 100 µM) for 2 h.

The cells were seeded into 6-well inserts with a polyethylene terephthalate (PET) membrane (pore size of 0.4 µm) (BD Falcon) at a density of 1.0 × 10^5^ cells. Cell culture was maintained at 37 °C under 90% humidity and 5% CO_2_. The medium was replaced every 2–3 days for both the apical (AP) and basolateral (BL) chambers of the transwell filters. Cell monolayers were used 19–21 days after seeding, once confluence and differentiation were achieved. To check the integrity of each cell monolayer, the transepithelial electrical resistance (TEER) was measured before and after the experiments with an epithelial voltohmmeter (Millicell-ERS^®^). Permeability studies were performed by adding apigenin and apigenin-K at a concentration of 50 µM dissolved in HBSS medium (pH 7.4). The transport experiment was initiated by removing the culture medium from the AP and BL chambers. HBSS medium was prewarmed to wash the Caco-2 monolayers twice and, subsequently, to incubate the monolayers for 30 min at 37 °C. Afterwards, the test compounds were added to the apical (AP) or basolateral (BL) chambers to perform bidirectional permeability studies, while the receiving chamber contained the corresponding volume of HBSS. The six-well plate containing the cell monolayers was put into an orbital environmental shaker, which was set at a constant temperature (37 °C) and agitation rate (54 rpm) for the duration of the permeability experiments. To follow transport across the cell monolayer, several samples of 200 µL each were collected at different time points (0, 30, 60, 90, and 120 min) from the AP or BL chambers during the permeability assay. The total volume in the chamber was maintained throughout the experiment, replacing the sample volume taken with an equal volume of HBSS. Moreover, two samples of 200 µL were taken from the donor chamber at the beginning and the end of the assay, for the mass balance calculation and validation of each replicate. The samples were centrifuged for 15 min at 15,000 rpm and 4 °C. The supernatants (cytoplasmic fraction) and the pellets (cell membranes) were stored at −80 °C until analysis was performed.

Transport studies were performed from apical-to-basolateral (AP-BL) and basolateral-to-apical (BL-AP) chambers. The apparent permeability (*P_app_*) values for each compound and direction can be calculated according to the following equation:

(2) $$P_{app}=\;\frac{dQ}{dt}\times \frac{1}{A\times C_{0}\times 60}$$

where *P_app_* is the apparent permeability (cm/s), *dQ*/*dt* is the steady state flux, *A* is the diffusion area of the membrane (cm^2^), *C*_0_ is the initial compound concentration in the donor compartment (µM) and 60 is a conversion factor [54]. The dilution suffered in the receiving chamber after each sample was taken into consideration.

In addition, the efflux ratio (*ER*) was calculated to determine the absorption mechanism, such as the ratio of *P_app_* (BL-AP) to *P_app_* (AP-BL).

(3) $$ER=\;\frac{P_{app}\;\left(\mathrm{BL}\text{-}\mathrm{AP}\right)}{P_{app}\;\left(\mathrm{AP}\text{-}\mathrm{BL}\right)}$$

Finally, as mentioned in the Discussion section, the diffusive flow of a substance in a medium, such as the epithelial barrier, depends on the concentration gradient of substance and the diffusion coefficient of the substance in the medium, as is expressed by Fick’s First Law:

(4) $$J=\;D\times \frac{dC\left(x\right)}{dx}$$

where *J* is the flow in the direction from the donor side of the barrier, *D* is the diffusion coefficient, *x* is the distance from the donor compartment, and *C*(*x*) is the concentration.

### 4.7. Analytical Methodology

The analyses of the samples from the permeability assay were analyzed via a high-performance liquid chromatograph Agilent LC 1100 series (Agilent Technologies, Inc., Palo Alto, CA, USA) controlled by the ChemStation software and equipped with a pump, autosampler, column oven and UV-Vis diode array detector. The samples were separated on a Poroshell 120 SB-C18 column (2.7 µm, 4.6 × 150 mm) after each 10 µL injection and were monitored at 280 nm. The mobile phases consisted of 0.1% TFA in water as mobile phase A and acetonitrile as mobile phase B, using the following multistep linear gradient: 0 min, 25% B; 5 min, 40% B; 10 min, 50% B; 15 min, 25% B; and 20 min, 25% B. The flow rate used was 0.5 mL/min and the column temperature was set at 22 °C. Quantitative evaluations of the apigenin or apigenin-K concentration were performed using the corresponding calibration graph of each compound with a six-point regression curve (r^2^ > 0.999). 

### 4.8. Statistical Analysis

Cellular data re expressed as the means ± SDs of six-to-ten replicates depending on the assay. One-way ANOVA and statistical comparisons of the different treatments were found using Tukey´s test for the photoprotection assay. In the rest of the assays, statistically significant differences were determined by applying the Student’s *t*-test. All analyses were performed in GraphPad Prism version 6.00 (GraphPad Software, San Diego, CA, USA). 

## Figures and Tables

**Table 1:** Values for different antioxidant measurements performed with apigenin and apigenin-K.

Antioxidant Assay (units)	Apigenin	Apigenin-K
TEAC (µmol TE ^a^/mmol)	2022.2 ± 154.8	1903.6 ± 210.5
FRAP (µmol Fe^2+^/mmol)	113.2 ± 12.2	88.7 ± 14.4 ***
ORAC (µmol TE ^a^/mmol)	887.9 ± 5.8	840.2 ± 28.3 *

^a^ Trolox equivalents. Values are expressed as the means ± SDs. * *p* < 0.05 and *** *p* < 0.001 indicate statistically significant differences between the antioxidant capacities measured for both apigenins.

**Table 2:** Apparent permeability (*P_app_*) values for apigenin and apigenin-K from apical-to-basolateral (AP-BL) and basolateral-to-apical (BL-AP) compartments.

Compound	*P_app_*_,AP-BL_ (× 10^−5^ cm/s)	*P_app_*_,BL-AP_ (× 10^−5^ cm/s)	Efflux Ratio
Apigenin	1.81 ± 0.17	0.93 ± 0.13	0.51
Apigenin-K	1.78 ± 0.21	0.92 ± 0.14	0.52

The data are expressed as the means of 6 replicates ± SDs.

## Supplementary Materials

Supplementary materials can be found at https://www.mdpi.com/1422-0067/20/9/2148/s1. Figure S1. Polyphenol (soft green) and flavonoid (dark green) classification (A) and apigenin basic structure and main biological effects (B).

## References

[1] Pullar J.M., Carr A.C., Vissers M.C.M. (2017). The roles of vitamin c in skin health. Nutrients.

[2] Madison K.C. (2003). Barrier function of the skin: “La raison d’etre” of the epidermis. J. Investig. Dermatol..

[3] Shindo Y., Witt E., Han D., Epstein W., Packer L. (1994). Enzymic and non-enzymic antioxidants in epidermis and dermis of human skin. J. Investig. Dermatol..

[4] Pérez-Sánchez A., Barrajón-Catalán E., Herranz-López M., Micol V. (2018). Nutraceuticals for skin care: A comprehensive review of human clinical studies. Nutrients.

[5] D’Orazio J., Jarrett S., Amaro-Ortiz A., Scott T. (2013). Uv radiation and the skin. Int. J. Mol. Sci..

[6] Nichols J.A., Katiyar S.K. (2010). Skin photoprotection by natural polyphenols: Anti-inflammatory, antioxidant and DNA repair mechanisms. Arch. Dermatol. Res..

[7] Lavker R.M., Gerberick G.F., Veres D., Irwin C.J., Kaidbey K.H. (1995). Cumulative effects from repeated exposures to suberythemal doses of uvb and uva in human skin. J. Am. Acad. Dermatol..

[8] Wlaschek M., Heinen G., Poswig A., Schwarz A., Krieg T., Scharffetter-Kochanek K. (1994). Uva-induced autocrine stimulation of fibroblast-derived collagenase/mmp-1 by interrelated loops of interleukin-1 and interleukin-6. Photochem. Photobiol..

[9] Ichihashi M., Ueda M., Budiyanto A., Bito T., Oka M., Fukunaga M., Tsuru K., Horikawa T. (2003). UV-induced skin damage. Toxicology.

[10] Sander C.S., Chang H., Hamm F., Elsner P., Thiele J.J. (2004). Role of oxidative stress and the antioxidant network in cutaneous carcinogenesis. Int. J. Dermatol..

[11] Brem R., Guven M., Karran P. (2017). Oxidatively-generated damage to DNA and proteins mediated by photosensitized UVA. Free Radic. Biol. Med..

[12] Sung B., Chung H.Y., Kim N.D. (2016). Role of apigenin in cancer prevention via the induction of apoptosis and autophagy. J. Cancer Prev..

[13] Zhou Y., Zheng J., Li Y., Xu D.P., Li S., Chen Y.M., Li H.B. (2016). Natural polyphenols for prevention and treatment of cancer. Nutrients.

[14] Chen X.J., Wu M.Y., Li D.H., You J. (2016). Apigenin inhibits glioma cell growth through promoting microrna-16 and suppression of BCL-2 and nuclear Factor-κB/MMP9. Mol. Med. Rep..

[15] Losada-Echeberria M., Herranz-Lopez M., Micol V., Barrajon-Catalan E. (2017). Polyphenols as promising drugs against main breast cancer signatures. Antioxidants.

[16] Perez-Sanchez A., Barrajon-Catalan E., Ruiz-Torres V., Agullo-Chazarra L., Herranz-Lopez M., Valdes A., Cifuentes A., Micol V. (2019). Rosemary (*Rosmarinus officinalis*) extract causes ROS-induced necrotic cell death and inhibits tumor growth in vivo. Sci. Rep..

[17] Thimoteo N.S.B., Iryioda T.M.V., Alfieri D.F., Rego B.E.F., Scavuzzi B.M., Fatel E., Lozovoy M.A.B., Simao A.N.C., Dichi I. (2018). Cranberry juice decreases disease activity in women with rheumatoid arthritis. Nutrition.

[18] Alvarez-Martinez F.J., Barrajon-Catalan E., Encinar J.A., Rodriguez-Diaz J.C., Micol V. (2019). Antimicrobial capacity of plant polyphenols against gram-positive bacteria: A comprehensive review. Curr. Med. Chem..

[19] Sun Y., Tao W., Huang H., Ye X., Sun P. (2019). Flavonoids, phenolic acids, carotenoids and antioxidant activity of fresh eating citrus fruits, using the coupled in vitro digestion and human intestinal HEPG2 cells model. Food Chem..

[20] Perez-Sanchez A., Barrajon-Catalan E., Caturla N., Castillo J., Benavente-Garcia O., Alcaraz M., Micol V. (2014). Protective effects of citrus and rosemary extracts on uv-induced damage in skin cell model and human volunteers. J. Photochem. Photobiol. B.

[21] Perez-Sanchez A., Barrajon-Catalan E., Herranz-Lopez M., Castillo J., Micol V. (2016). Lemon balm extract (*Melissa officinalis*, L.) promotes melanogenesis and prevents uvb-induced oxidative stress and DNA damage in a skin cell model. J. Dermatol. Sci..

[22] Nobile V., Michelotti A., Cestone E., Caturla N., Castillo J., Benavente-Garcia O., Perez-Sanchez A., Micol V. (2016). Skin photoprotective and antiageing effects of a combination of rosemary (*Rosmarinus officinalis*) and grapefruit (*Citrus paradisi*) polyphenols. Food Nutr. Res..

[23] Mink P.J., Scrafford C.G., Barraj L.M., Harnack L., Hong C.P., Nettleton J.A., Jacobs D.R. (2007). Flavonoid intake and cardiovascular disease mortality: A prospective study in postmenopausal women. Am. J. Clin. Nutr..

[24] Knekt P., Kumpulainen J., Jarvinen R., Rissanen H., Heliovaara M., Reunanen A., Hakulinen T., Aromaa A. (2002). Flavonoid intake and risk of chronic diseases. Am. J. Clin. Nutr..

[25] Lefort E.C., Blay J. (2013). Apigenin and its impact on gastrointestinal cancers. Mol. Nutr. Food Res..

[26] Formica J.V., Regelson W. (1995). Review of the biology of quercetin and related bioflavonoids. Food Chem. Toxicol..

[27] Tang D., Chen K., Huang L., Li J. (2017). Pharmacokinetic properties and drug interactions of apigenin, a natural flavone. Expert Opin. Drug Metab. Toxicol..

[28] Bak M.J., Das Gupta S., Wahler J., Suh N. (2016). Role of dietary bioactive natural products in estrogen receptor-positive breast cancer. Semin. Cancer Biol..

[29] Salinas J.L., Sánchez J.C., Garcia O.B.-G., Ortega V.V., Gascón J.Y., Muñoz F.S., Baños M.A., Borrón J.C.G., Teruel J.A.L. (2004). Use of Compounds Derived from 2,3-Dehydronaringenin for the Treatment of Inflammatory Processes and Pharmaceutical Composition Containing Said Derivatives. U.S. Patent.

[30] Amidon G.L., Lennernas H., Shah V.P., Crison J.R. (1995). A theoretical basis for a biopharmaceutic drug classification: The correlation of in vitro drug product dissolution and in vivo bioavailability. Pharm. Res..

[31] Madunic J., Madunic I.V., Gajski G., Popic J., Garaj-Vrhovac V. (2018). Apigenin: A dietary flavonoid with diverse anticancer properties. Cancer Lett..

[32] Shukla S., Gupta S. (2010). Apigenin: A promising molecule for cancer prevention. Pharm. Res..

[33] Cushnie T.P., Lamb A.J. (2005). Antimicrobial activity of flavonoids. Int. J. Antimicrob. Agents.

[34] Mascaraque C., Gonzalez R., Suarez M.D., Zarzuelo A., Sanchez de Medina F., Martinez-Augustin O. (2015). Intestinal anti-inflammatory activity of apigenin k in two rat colitis models induced by trinitrobenzenesulfonic acid and dextran sulphate sodium. Br. J. Nutr..

[35] Guerrero L., Castillo J., Quiñones M., Garcia-Vallvé S., Arola L., Pujadas G., Muguerza B. (2012). Inhibition of angiotensin-converting enzyme activity by flavonoids: Structure-activity relationship studies. PLoS ONE.

[36] Huang D., Ou B., Prior R.L. (2005). The chemistry behind antioxidant capacity assays. J. Agric. Food Chem..

[37] Benzie I.F., Strain J.J. (1996). The ferric reducing ability of plasma (Frap) as a measure of “antioxidant power”: The frap assay. Anal. Biochem..

[38] Matthäus B. (1998). Isolation, fractionation and hplc analysis of neutral phenolic compounds in rapeseeds. Food/Nahrung.

[39] Montes de Oca M.K., Pearlman R.L., McClees S.F., Strickland R., Afaq F. (2017). Phytochemicals for the prevention of photocarcinogenesis. Photochem. Photobiol..

[40] Duke J.A., Beckstrom-Sternberg S.M. (2000). Handbook of Medicinal Mints (Aromathematics): Phytochemicals and Biological Activities.

[41] Rice-Evans C.A., Miller N.J., Paganga G. (1997). Antioxidant properties of phenolic compounds. Trends Plant Sci..

[42] Tabart J., Kevers C., Pincemail J., Defraigne J.O., Dommes J. (2009). Comparative antioxidant capacities of phenolic compounds measured by various tests. Food Chem..

[43] Eaton E.A., Walle U.K., Lewis A.J., Hudson T., Wilson A.A., Walle T. (1996). Flavonoids, potent inhibitors of the human p-form phenolsulfotransferase. Potential role in drug metabolism and chemoprevention. Drug Metab. Dispos..

[44] Wu S., Xu W., Wang F.R., Yang X.W. (2015). Study of the biotransformation of tongmai formula by human intestinal flora and its intestinal permeability across the Caco-2 cell monolayer. Molecules.

[45] Teng Z., Yuan C., Zhang F., Huan M., Cao W., Li K., Yang J., Cao D., Zhou S., Mei Q. (2012). Intestinal absorption and first-pass metabolism of polyphenol compounds in rat and their transport dynamics in Caco-2 cells. PLoS ONE.

[46] Tian X.J., Yang X.W., Yang X., Wang K. (2009). Studies of intestinal permeability of 36 flavonoids using Caco-2 cell monolayer model. Int. J. Pharm..

[47] Fang Y., Cao W., Xia M., Pan S., Xu X. (2017). Study of structure and permeability relationship of flavonoids in Caco-2 cells. Nutrients.

[48] del Mar Contreras M., Borrás-Linares I., Herranz-López M., Micol V., Segura-Carretero A. (2016). Further exploring the absorption and enterocyte metabolism of quercetin forms in the Caco-2 model using nano-LC-TOF-MS. Electrophoresis.

[49] Liu L., Guo L., Zhao C., Wu X., Wang R., Liu C. (2015). Characterization of the intestinal absorption of seven flavonoids from the flowers of trollius chinensis using the Caco-2 cell monolayer model. PLoS ONE.

[50] Ravisankar S., Agah S., Kim H., Talcott S., Wu C., Awika J. (2019). Combined cereal and pulse flavonoids show enhanced bioavailability by downregulating phase ii metabolism and abc membrane transporter function in Caco-2 model. Food Chem..

[51] Alvarez A.I., Real R., Perez M., Mendoza G., Prieto J.G., Merino G. (2010). Modulation of the activity of abc transporters (P-glycoprotein, MRP2, BCRP) by flavonoids and drug response. J. Pharm. Sci..

[52] Zhang J., Liu D., Huang Y., Gao Y., Qian S. (2012). Biopharmaceutics classification and intestinal absorption study of apigenin. Int. J. Pharm..

[53] Barrajon-Catalan E., Fernandez-Arroyo S., Saura D., Guillen E., Fernandez-Gutierrez A., Segura-Carretero A., Micol V. (2010). Cistaceae aqueous extracts containing ellagitannins show antioxidant and antimicrobial capacity, and cytotoxic activity against human cancer cells. Food Chem. Toxicol..

[54] Lin H., Gebhardt M., Bian S., Kwon K.A., Shim C.K., Chung S.J., Kim D.D. (2007). Enhancing effect of surfactants on fexofenadine HCL transport across the human nasal epithelial cell monolayer. Int. J. Pharm..

