# Supplementary figures and images for: Antioxidant and Photoprotective Activity of Apigenin and Its Potassium Salt Derivative in Human Keratinocytes and Absorption in Caco-2 Cell Monolayers
Source: Int J Mol Sci. 2019 Apr 30;20(9):2148. doi: 10.3390/ijms20092148 (PMC6539602; doi:10.3390/ijms20092148)

**A**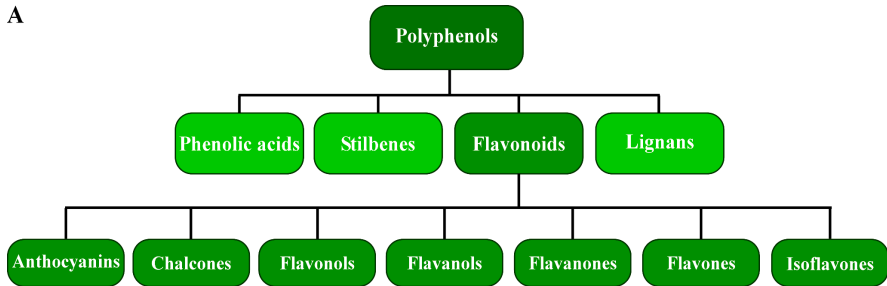**B**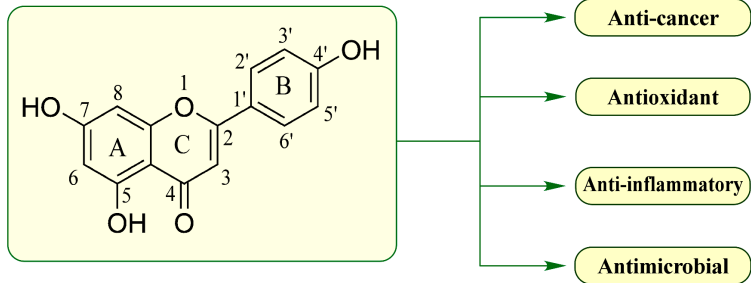

Supplement: Supplementary file 1 [file ijms-20-02148-s001.pdf]
